# Mental Health, Declining Physical Activity and Social Connection during Transitions into Fatherhood in the UK

**DOI:** 10.3390/ijerph21070890

**Published:** 2024-07-09

**Authors:** Emily Lovett, Andy Smith

**Affiliations:** Centre for Mental Health, Sport and Physical Activity Research, Edge Hill University, Ormskirk L39 4QP, UK

**Keywords:** fathers, life transitions, physical activity, loneliness, mental health, perinatal

## Abstract

This paper addresses an under-explored area of sociologically oriented health research, namely, the mental health and physical activity (PA) experiences of new fathers. Drawing upon responses to an online qualitative survey from 32 fathers, aged 18 or over, and living in the UK, we show how the decline in these fathers’ overall PA was associated with poor mental health and the changing constraints that characterised their increasingly complex networks of interdependence. These constraints corresponded with shifts in fathers’ PA engagement from team sports towards individualised, flexible, and more recreationally oriented lifestyle activities like running and the gym. Fathers’ engagement in these activities appeared to exacerbate the complex feelings of guilt and isolation that they already encountered as new fathers. These experiences were simultaneously associated with feelings of shame associated with being insufficiently active and fearing judgement about their engagement in fathering responsibilities. The paper has important policy implications, highlighting the need for tailored support for new fathers in the perinatal period, and implications for practice, suggesting that co-produced community-based PA programmes are potentially effective settings for engaging new fathers in PA and promoting their mental health.

## 1. Introduction

There is increasing international interest in perinatal mental health (i.e., mental health from conception to the end of the first postnatal year), which is an umbrella term often used to encompass ‘mental health problems, psychological distress and psychological wellbeing’ [1]. However, much of the existing research focuses on maternal mental health, including during the perinatal period [1,2]. Relatively little is known by comparison about fathers’ mental health, despite paternal stress rising (sometimes substantially so) during the perinatal period and especially around the time of birth [2]. It is also estimated that 5–10% and 5–15% of fathers experience perinatal depression [3] and anxiety [4], respectively, and up to one-quarter of new fathers are thought to experience postpartum depression during the first 12 months after birth [5].

The long-term costs of perinatal mental illness are significant. For mothers, in 2014, it was estimated that perinatal depression, anxiety, and psychosis had a long-term cost of GBP 8.1 billion for each one-year cohort of births in the UK (72% of which related to impacts on the child), with GBP 1.2 billion of those costs being borne by the National Health Service and social services [6,7]. In the United States, the costs of untreated perinatal mood and anxiety disorders are also increasing, with estimates suggesting that these conditions cost USD 14 billion for the 2017 birth cohort from conception to five years postpartum [8]. As well as having substantial costs for the lives of mothers (including reduced ability to pursue paid employment and engage in other activities), there are ‘well-established adverse impacts of untreated perinatal mental health problems on pregnancy outcomes, infant growth and development, and offspring educational attainments’ [9]. To these can be added increased lifetime health service use, reduced quality of life, and widening already increasing inequalities in the social determinants of mental health (e.g., poverty, income inequality, job insecurity, housing), which disproportionately impact the long-term wellbeing of those with poorer mental health [10,11,12]. 

Despite their prevalence and adverse impacts, in many countries, ‘most perinatal mental health problems remain unidentified and untreated’ [9], and the costs of paternal perinatal mental illness are currently unknown [1]. There also remains ‘a dearth of information regarding men’s experiences of their own perinatal mental health, and our understanding of how best to address fathers’ mental health and psychological wellbeing…is severely limited’ [1], including in relation to physical activity (PA). In this paper, we seek to address this lacuna in knowledge by exploring the mental health experiences of first-time fathers and fathers who have additional children and how these experiences impacted their engagement in PA. Specifically, we sociologically explore fathers’ mental health and PA during the perinatal period and in the context of their transitions to parenthood until their children were aged four years, using novel evidence from an online survey of fathers living in the UK. Our findings reveal how declining PA for new fathers is a normalised but isolating feature of transitions into parenthood or when caring for babies and young children. Fathers’ experience of guilt, shame, and isolation is also explored. The findings are important for policy and practice, as we shed new light on the potential of PA as an important context in which to promote the mental health of new fathers by, among other things, tackling social isolation and providing peer support. 

### 1.1. Transitions into Parenthood and Fathers’ Mental Health 

There is a growing body of evidence suggesting that transitions into parenthood can be associated with increased risk of mental illness [1,13,14,15], stress [1,13,14,15], and a correlative reduction in PA [16,17] as the relational constraints associated with becoming a parent lengthen and become more complex [18,19]. For fathers, additional stressors associated with transitions to parenthood include changing role restrictions, lifestyles, and relationships [15,20,21], loneliness [22], and new challenges with maintaining a positive work–life balance [14]. Others have identified some fathers’ negative feelings about, or fear of, pregnancy, childbirth, and incompetence in infant care as contributing to paternal stress and an increased risk of anxiety, depression, psychological distress, and fatigue [23]. These typically result in what is commonly described as a rollercoaster of feelings associated with becoming a father [15,20].

Although current work has recognised the importance of intimate fatherhood [24], ‘involved fathering’ [25,26], and caring masculinities [27] for mental health, sometimes fathers are described as seeing themselves as being ‘on the inside looking in’ or as ‘present, but not participating’ [28] in parenting. Clearly, there are tensions between traditional role expectations about men and fathers and contemporary involved fathering ideals that many men are actively engaged in [29]. The marginalisation of some fathers during the perinatal period where they are positioned as a supporter, rather than as somebody to be supported or involved, yet unimportant [29], can compromise fathers’ mental health. Dolan and Coe [30] have noted how some health care professionals (HCPs) contribute to that marginalisation by positioning them on the side-lines of perinatal care and reproducing perceptions and ideals about dominant masculine identities. Tarrant [31] has similarly suggested that many perinatal services remain heavily gendered, often to the detriment of fathers’ mental health, while Baldwin et al. [20] have argued that antenatal classes typically focus on the needs of women, and many men can thus feel uncomfortable asking for support in perinatal services, breastfeeding support groups, and mother and baby classes. Given their dominant positioning as stoic supports, many men are said to develop what Hodkinson and Das [29] call ‘repertoires of illegitimacy’, where maternity services are believed to be already in significant demand and should focus particularly on the needs of mothers.

In their qualitative study of new fathers’ perceptions of the support that they received during the perinatal period, Hambidge et al. [2] reported that over one-half of their respondents had experienced mental illness for the first time in the year after the birth of a child. This was typically associated with feelings of shame, and many of the participants were offered no support, as HCPs focused on the mother and baby [2]. Fathers have also suggested that it can feel socially and culturally unacceptable and shameful to speak negatively about fatherhood and discuss experiencing mental health difficulties [20,32]. Shame is often experienced because not only of a negative perception of oneself, but also of the judgement or the anticipated judgement of others [33]. In considering Elias and Scotson’s [34] explanations of established and outsider relations, where outsider groups are often stigmatised through historical and social processes between groups with uneven power balances, Doblytė [35] explained that stigma, self-stigma, and the anticipation of shame can impede or delay healthcare seeking for mental illness. For new fathers who are already feeling like the ‘outsider’ group in maternity services, stigma and feelings of shame are thus often central to many of their thoughts and feelings about perinatal support services. 

### 1.2. Supporting New Fathers’ Mental Health 

Parental mental illness, especially that experienced by fathers, increases the risk of child morbidity and mortality [36], and this emphasises the importance of supporting families who face adversity during the first 1001 critical days (conception to age 2) to increase the likelihood of a child reaching their potential [11,37]. Supporting parental mental health can improve parents’ ability to bond with their baby and create the secure attachments vital for infant mental health [37], ensuring every child gets the best start in life [11], as well as helping to reduce public spending on supporting children later in life [11,37]. Engaged fatherhood is also increasingly viewed as a central tenet of citizenship for men [21] that has long-term benefits for child development and mental health [37] and can be strengthened by improving fathers’ experiences of perinatal support services [29,31,38,39]. Exploring new ways to engage new fathers and include minoritized fathers in those services and wider networks of informal support is critical [14,31,40]. This is particularly important, given that some fathers have explained how friends who are not parents are less likely to relate to their parental and associated mental health challenges, whilst connections made with other fathers through networks from antenatal classes, though they can be helpful, often remain light-hearted, rather than deep and meaningful [20]. Another source of support for mental health during the transition into parenthood is engaging in PA. 

### 1.3. Physical Activity, Mental Health, and Transitions into Parenthood 

Given that many men do not access traditional sources of support for their mental health, it is important to explore other informal activities and settings. These settings include non-clinical community assets (e.g., professional sports club stadia, cultural organisations), which have been shown to be important, culturally acceptable ways of engaging men in PA and mental health promotion programmes [41,42,43]. Such programmes, which employ non-stigmatising language and enable men to engage in interactive, non-threatening, and solutions-focused activities, are critical to increasing PA and improving mental health [41,42,43,44]. More broadly, PA and the closely related concept of exercise have well-reported benefits for mental health [45,46], and evidence suggests that they can be used for both the prevention [47,48] and the treatment [46,48,49,50] of mental illness. Regular PA and exercise—including alongside talking therapies—are recommended self-help strategies for some mild to moderate perinatal mental illnesses, including postnatal depression, perinatal anxiety, PTSD, and birth trauma [51,52]. Having noted the decline in PA in fatherhood, Young and Morgan [17] stress the need for high-quality research examining interventions engaging fathers in personal and family-based interventions, using PA for benefiting the fathers’ physical and mental health, fostering bonding between the father and their child(ren), and benefiting the child’s health. As PA (including through sport) is viewed as an important space for engaging many men, it is important to explore men’s experiences of the relationship between PA and mental health during transitions into fatherhood.

Despite the mental health benefits of PA and exercise and the well-reported general declines in PA from mid-adolescence and during complex life transitions including becoming a parent [53,54]—where parents act as key socialisation agents into physical activity but, often, by becoming the providers of activity for children, rather than participants [16]—relatively little is known about new fathers’ engagement in PA and its relationship with mental health. In the rest of this paper, therefore, we address this important gap in knowledge by presenting new evidence on the mental health and PA experiences of new fathers or of fathers of babies and young children, as they transition into parenthood and through the early years. Exploring this from a sociological perspective helps us to move away from understanding fathers and their experiences of PA and mental health in highly individualized, reductionist ways, which typically characterises more quantitative or psychological studies. Instead, locating fathers and their experiences in the complex and dynamic interdependencies of family life and, more broadly, of their social relations is a more adequate way of conceptualizing fathers’ mental health and engagement in PA as inherently relational, processual, and embodied biopsychosocial phenomena.

## 2. Materials and Methods

The evidence presented in this paper was derived from a larger survey of 218 new parents and reports on the mental health and PA experiences of 32 fathers (representing 14.7% of all survey respondents) who responded to a qualitative online survey. This method has the potential to generate rich and broad data [55] on fathers’ transitions into parenthood, PA, and mental health. An anonymous survey provided fathers in a participant group who might be experiencing undiagnosed mental illness or shame relating to poor mental health or mental illness in the perinatal period [2] with an opportunity to explain their experiences openly without fear of judgment (the participants were able to voluntarily provide their email address at the end of the survey if they wished to). 

### 2.1. Participants 

This qualitative online survey was open to all parents in the UK with children up to four years of age. This included the perinatal period (from conception to one year after birth) and beyond, so that the parents were able to reflect on changes in their mental health and PA over that period. Online surveys shared through networks and social media rely on participants to opt-in. It was notable that when the survey was promoted to ‘new parents’, the initial response from new fathers was very low. Taking advice from charities working specifically with new fathers, survey promotion material was adapted to specifically target fathers, and this increased the response rate to 32 fathers. Their lack of engagement in a ‘parent’ survey compared to a ‘dad’ survey was telling. As Table 1 indicates, of the 32 men who completed the survey, 91% were white, 78% were between 30 and 39 years of age, 81% were in opposite-sex married-couple families, 94% were in full-time employment, none disclosed any disability, and 34.5% had/maybe experienced mental illness. 

### 2.2. Procedure 

Having gained institutional ethical approval (SPA-REC-2018-342), Jisc Online Surveys (formerly BOS) was used to design and distribute the surveys. The surveys consisted of 22 questions and generated basic quantitative demographic data (9 questions) and data on time and type of PA engagement and experience of mental health (currently, in the past, and maybe undiagnosed) (3 questions). More detailed qualitative data were also generated (10 questions plus sub-questions) on the respondents’ experiences of mental health and PA, PA behaviours, and PA behaviours in the context of family life. Example questions included the following: ‘what (if anything) makes staying physically active difficult? Please describe your experiences and feelings in as much detail as possible’; ‘please describe how you feel about being physically active with or without your child(ren) present’; and ‘have you used (or are you using) physical activity for your mental health?’. The respondents were also asked to describe any other support (if any) they sought for their mental health. Advice to seek support from a health care professional and links to appropriate support services were provided at the end of the survey. 

The survey was made available from May 2020, but promotion via networks with local and national charities and on social media was paused until April 2021 because of the COVID-19 pandemic and associated national lockdowns in the UK. The survey remained open until the end of September 2021. Whilst there was an easing of restrictions when most participants completed the survey, some fathers with young children might have had babies during periods of national lockdown when restrictions in maternity services were still in place and likely to exacerbate fathers’ tendency to exist on the periphery of those services [29,56]. 

### 2.3. Data Analysis 

Jisc Online Surveys was used to produce and record the basic descriptive quantitative data from the closed questions. NVivo 12 was used to help manage the qualitative data to ease the process of coding and data retrieval as the researchers engaged in reflexive thematic analysis (see [57]). Except for any self-evident typographical errors, the text was analysed as written by the participants who were assigned pseudonyms, which are used when presenting their responses below. 

Using Braun and Clarke’s [57] six-phase reflexive thematic analysis, the analysis and coding of the qualitative data were undertaken by the first author. Phase 1 (familiarization), involved a detailed reading and re-reading of the responses provided. In phase 2, initial codes (e.g., ‘isolation’, ‘less activity’, ‘interaction’, ‘guilt’, ‘loneliness’) were generated. Candidate themes were then constructed in Phase 3 by bringing together clusters of codes (e.g., ‘mental health and fathers’ isolation’, ‘guilt, loneliness, and declining PA’). The candidate themes were constructed to reflect the patterns of shared meaning perceived to exist in the data [57]. These candidate themes were first discussed with the second author and then between the authors with colleagues during informal research group meetings. In Phase 4, themes were developed and reviewed, checking that they made sense in relation to the whole dataset. The themes were refined by the co-authors in Phase 5, as final reflexive, or fully realised, themes were generated and named considering the existing sociological and other research on parental mental health and PA. The final, fully realised, themes were generated through writing up the research and further discussion about prioritising content (phase 6) and are the following: (i) declining physical activity as a normalized but isolating feature of becoming a parent; (ii) guilt as a central and guiding emotion; (iii) reconnecting through physical activity with ‘people like me’. 

### 2.4. Researcher Reflexivity

Emily Lovett is a mother of two children with experience of sociological research in community sport, physical activity, and mental health promotion. Emily was the project lead and worked with local and national parenting and perinatal mental health charities to design and promote the survey. She also undertook initial coding and coordinated discussions for further analysis of the data. Andy Smith is a professor in sport, PA, and mental health with experience of working with fathers with poor mental health in community settings and contributed to survey design, reflexive thematic analysis, and paper writing. Neither researcher had lived experience as a father, but as sociologists, it remained important for them to continually check and challenge their involvement and detachment in the research and research process throughout the project. 

## 3. Results and Discussion

### 3.1. Declining PA as a Normalized but Isolating Feature of Becoming a Parent 

Two-fifths of the fathers (40%) reported that they were meeting the recommended 150 min of PA per week, and 81% said that they were using PA for mental health benefit; most fathers reported their PA declined during the transition into parenthood and that they would like to be more active. The reasons for the decreasing PA were associated with fathering responsibilities and how this affected them, including increased feelings of isolation during their transition into parenthood. For example, Paul explained:

I have drastically reduced my participation, and it now all revolves around family. I had always coached football which made up for having to stop playing and helped keep me fit to some degree however, I have recently stopped this due to commitments of having a second child. It can breed some resentment at home which I even feel guilty about.

This was also experienced by Robert who wrote that he had ‘reduced time and therefore exercise always comes last. Less activity which makes me feel lethargic, tired’ as he sought to balance his time between being active and the increasing constraints associated with parenthood [19]. Indeed, other fathers referred to other significant parental constraints on their ability to be active, including time (Mark, Phil, and Gareth), work (Martin and Dan), and commitments and responsibilities (Robert, Jacob, Adrian, and Luke). Whilst fathers have traditionally been identified as playing the provider role in families, there has been a gradual shift towards ‘involved fathering’ [24,25,26]. For Kay [26], there are multiple identities of fatherhood that are not necessarily compatible, since many fathers are often trying to balance expectations of caregiving through their involvement in the home and family life with providing financially through their involvement in the labour market. The complexities of these varied demands and increased relational constraints require adjustment for new fathers. In discussing the rejection of domination in masculine identities towards caring masculinities, Elliot [27] highlighted the relational and interdependent qualities of care that can be embraced alongside positive emotion. The fathers in our study clearly had a propensity towards caring or the feeling of responsibility at home, but also wished to remain active. In trying to balance their commitments and their desire to remain active, many of the new dads in this study altered their activity. Mike and Tim curtailed their involvement in team sports in favour of more flexible, less time-bound, individual activities that could be accommodated more easily within their busy schedules and without the need to engage with teammates [53,54]. Mike explained his experience of this as follows:

I used to play a lot more sport such mainly football. As I have had children, I find being away for half a day on a Saturday and committing to that regularly has been difficult. Also picking up injuries would then have an impact on my home life which makes me more hesitant to play as much intense sport. So I have started gym classes which I never used to do before children.

Tim also described how he does not engage in ‘longer activities like playing football or golf’ anymore but instead engages in activities that are ‘short and sharp and fit in between many things, or at the very end of the day when I am already knackered’. The social interaction that accompanied his PA was described by Tom as follows:

I previously could choose to do what I want when I want. The sports I do are largely combined with social interaction and sometimes it’s tricky to juggle my own, sometimes limited, schedule with others.

Some were already engaged in individual activities like running, but most fathers who were already feeling increasingly isolated altered their PA to engage in more individual activities. This served to limit their social interactions with people whom they previously shared a connection with. 

The caregiving fathers interviewed by Hodkinson and Brooks [38] remained committed to their counter-normative caregiving roles but found some persistent gendered limitations (communication with institutions, networks with other parents, and maternal decision making and responsibility) in their fatherhood journeys. Transitions into fatherhood can be isolating, with a disconnection from friends and family outside of the home, and much of the attention, support, and medicalised focus is on the birthing mother and baby [29]. A sense of being different to other parents can also exacerbate feelings of disconnection or loneliness for stay-at-home fathers [22]. Whilst they were predominantly in full-time work, the fathers in our study echoed these feelings of isolation and loneliness. Matthew wrote that he felt ‘play groups targeted at mums and classes filled with mums singing songs about mums is really unhelpful and is a viscous cycle of leaving dads out of family units which has all kinds of impact’. Will also explained how

it feels quite lonely as a dad in the first year or so. My wife had various mum & baby classes she would go to and meet other parents during the day. Whereas I would be at work then come home and be straight into taking over with baby duties. It meant a period of time where any social interaction was minimal, especially with friends who didn’t have a family.

These men appeared to be experiencing ‘outsider’ feelings when describing their exclusion from baby groups while their social ties with their own friends were also diminishing. The wide spectrum of loneliness can include being surrounded by people, but with no affective meaning with those people [58], and unless new fathers—including those in this study—have meaningful connections or relatable experiences with the people that they interact with, feelings of loneliness are often encountered. Such feelings often result from the unintended outcomes of fathers’ reduced engagement in PA and its associated networks (especially for those involved in team sports) and include the ostensibly paradoxical feelings of isolation that accompany the lengthening and increasingly complex independency networks [19]. Increasingly involved fathers experience the various new and changing constraints in their familial relationships that often occur when becoming a parent and which, as the next section indicates, can result in increased feelings of guilt when trying to engage in PA.

### 3.2. Guilt as a Central and Guiding Emotion 

Guilt, experienced and reported in various ways by fathers, was identified as a central and guiding emotion. Such guilt emanated from fathers’ own expectations and the constraints they internalized from the perceived social expectations of those around them, including in relation to neoliberal ideals associated with being sufficiently physically active for health; the need to experience positive mental health for the benefit of one’s offspring; and the need to be present with family in caring roles. In relation to not being physically active enough, Adrian explained that ‘it’s really hard. I don’t do anywhere near enough. I do much less now than I ever did before. I don’t feel good about it’. Paul also recalled his feelings of disappointment of not being able to exercise thus: ‘I feel positive afterwards but can feel very disappointed with myself when I don’t manage to exercise’. These men appeared to have internalized the idea that they should be, and want to be, active and that in being insufficiently active they felt that they were somehow failing.

The tendency for fathers to internalize dominant social expectations about the need to adopt physically active lifestyles was also bound up in the tensions of a whole range of other social constraints [18] associated with the guilt reported by our respondents. These feelings of guilt included fathers’ desire to support their family and taking time away from family to be active. For example, Simon did not like ‘feeling as though I am leaving my wife on her own to look after child’ or ‘missing out on bonding with child’. This was echoed by Matthew who wrote:

Guilt. I don’t like feeling like I’m not there for my daughter and family or I can’t help if things get too much with our daughter. Opportunity to be active while both of us parents work and also do extra curricular work as well leads to busy lives which isn’t easy for me to commit to regular formal or informal activity. I go on a more ad hoc basis—and often at a point when things have got too much. Cost is also a barrier as I don’t feel like I should spend money on myself if it isn’t a family activity. When we were pregnant and when our daughter was very young I always wanted to be nearby in case help was needed or something happened.

Chris also described how he tried to ‘compensate in other areas to feel less guilty about having own exercise time, so try and make sure to do fair share of wake ups and bath and bed and be around during the day to help when needed’. Together with the guilt that the fathers felt about taking time and money away from family life for their own leisure-time PA, was a fear of being judged for doing so. Andrew expressed his concern about how he would be ‘seen’ when there were ‘jobs around that house that need doing’, writing that he did not ‘want to be seen wasting time that I could do them doing physical activity’. Trying to strike the balance, Mark explained how running regularly helps him to be ‘better able to concentrate when working’ and his ‘temperament is calmer and easier to control’, noting that without exercise, his ‘mental health suffers. It brings on anxiety that then makes me not want to exercise, which creates a cycle’. In explaining how he is supported with his PA, he explained that his partner ‘allows me time’ but also described some perceived expectations of him, suggesting that he ‘would like to swim more (open water if possible) but I already take the p*** with the amount of time I get to go running. If I added more to it I’ll be living alone!’.

Fear of judgement, as in Mark’s and other fathers’ account, is a key element of Scheff’s [33] explanations of shame, for which guilt is an emotion linked to seeing oneself negatively. Scheff [33] (p. 254) explains how guilt or shame can be experienced by both seeing oneself negatively ‘through the eyes of others, or only anticipating such a reaction’, as the fathers did in this study when discussing the guilt they experienced in relation to family time or their engagement in PA. Based on their feelings of guilt experienced by missing out on family bonding and fear of judgement by others, the fathers’ expressions of resentment at home appeared an effect of shame and anger, with the ‘anger pointed outward’, while guilt appeared to be experienced through that ‘anger pointed back at self’ [33] (p. 255). In discussing the changing expectations of fatherhood over time, Dermott [24] tried to move away from the dichotomous thinking of ‘involved’ versus ‘uninvolved’ fathers, preferring the consideration of intimacy in father–child relationships. Despite this, a culturally embedded ideal of the involved father was emerging and this, for Dermott, cited by Kay [26] (p. 17), was ‘creating new benchmarks by which fathers are judged’. Language about the judgement of fathers may perpetuate their positioning as the outsider group. Our behaviour, judgements, and fear of judgement remain inherently social. The social constraints, guilt, and anxiety surrounding behaving in a socially desirable way was clear from the fathers in this study who were juggling myriad relational constraints relating to work, home life, chores, and family bonding that competed for time and energy alongside their desire to remain physically active and mentally well. 

### 3.3. Reconnecting through Physical Activity with ‘People like Me’ 

Around one-third (*n* = 11; 35%) of the respondents reported either that they were (or maybe were) currently experiencing or that they had previously experienced mental illness in the perinatal period, but none of them described accessing support for their mental health. When asked what had helped or what they were doing to manage their mental illness, the fathers reported the following: sport/exercise/PA when they could fit it in (Will, Paul, Ben, Tom, Matthew, Harry, Tim), ‘time to myself’ (Paul) or ‘me time’ (Tim), ‘talking to my wife about my worries’ (Ben), ‘reduce alcohol’ (Ben), ‘trying to lower workload at job’ (Matthew), ‘music’ (Tim), or ‘nutrition and meditation (when I can make myself sit long enough)’ (Gareth). None of the fathers mentioned accessing any formal support for their mental health, which is often associated with a sense of shame and stigma that can result from fathers’ experiences as an outsider group [35]. This may be exacerbated in a group of men, including new fathers, who are often positioned as being on the periphery of perinatal mental health support services, which are most commonly thought to be most important for mothers [29].

Having found time for family chores and work by reducing their PA and socialising, some fathers wished to reconnect with PA, their peers, and their families. This was often to ‘stay healthy, enjoyment, good use of free time’ (Tom), the dads wanted to have ‘fun’ (Tim). Indeed, as advocated by Young and Morgan [17], family-based interventions for physical activity could potentially be used to strengthen bonds between the father and the child and play an important role in benefiting the physical and mental health of those involved. The desire to make the best use of their limited free time [59] was particularly important for the fathers in this study, with Chris suggesting that, until his child did not want to sit still for long enough, running with the buggy had the ‘dual benefit’ of being ‘good to give mum a break’. Matthew similarly suggested that he ‘would love to be active with my daughter’ and ‘missing out on bonding with child’ was mentioned as a barrier to PA for Simon.

Whether it was family-based PA or other ways of being active, the fathers were keen to develop social connections with ‘other new Dads’ in ‘non-clinical settings’ (Simon). Matthew suggested that a ‘local offering would mean that making local friends could have other benefits socially or for support’. In the first theme, we outlined declining PA as a normalised but isolating feature of the transition into fatherhood. New fathers were altering their activity to more efficient and more individual PA, thus limiting their social ties even further. We have seen from the literature and the data presented here that new fathers’ mental health and PA are impacted during the transitions into parenthood. Whilst new fathers may develop ‘repertoires of illegitimacy’ [29] and not access support, developing father-inclusive practice [39] and engaging with men in creative ways (engaging with men in community spaces, building quality relationships to work alongside men) can enable signposting to mainstream or other community-based services [31]. There is potential to help support new fathers’ mental health via PA when this fits into their new routines; it can be an efficient and effective use of time; and it is perceived to benefit their family. The benefits of affective ties, shared meaning, and common ideas in uniting people are important counters to the feelings of loneliness and isolation [58]. In tackling the way that fathers are often excluded from a lot of baby groups, Tim emphasised how ‘common goals and people with my experiences would also help’ and he also suggested the benefits of an informal group through ‘Facebook’ or ‘WhatsApp’ to help him to ‘stay connected’ with the activities and join when available, stating that ‘other people is [are] important’. Phil also felt that a ‘set time with others’ would encourage him’ but that needed to ‘fit around childcare and work’. Ben and John were very clear in their need to ‘chat to like-minded people’ (Ben) and share ‘lived experiences from ‘people like me’’ (John), whether this was a part of family, parent, and child activities or of activities with other adults. As these men were not previously accessing support for their mental health, community-based PA is a potentially important and effective setting in which to promote fathers’ mental health and engagement in appropriate peer support.

## 4. Conclusions

In this paper, we sought to address a hitherto under-explored area of sociological research, namely, the mental health and PA experiences of new fathers or of fathers of babies and young children as they transition into parenthood and throughout the early years. We have shown how, for the fathers in our study, declines in their overall PA was associated with poor mental health and the changing constraints which characterised their lengthening and increasingly complex networks of interdependence. These constraints also corresponded with a shift in the kinds of activities in which some fathers were able to engage, namely, largely individual, flexible, and more recreationally oriented lifestyle activities like running and attending the gym. Fathers’ increased engagement in these types of highly individualised activities appeared to exacerbate the feelings of guilt and isolation that they were already encountering as new fathers. These experiences were simultaneously encountered with mental health-related feelings of shame associated with being insufficiently physically active and fearing judgement about their engagement in fathering responsibilities.

This study provides new empirical data from fathers about their experiences of PA and mental health. However, the data generated in this study were predominantly from white men, living in opposite-sex married-couple families, who were employed in full-time work, and most likely had an interest in the topic to take part in an opt-in survey. It will be important for future research to examine more diverse father populations. It must also be recognised that whilst the survey was promoted after the national lockdown restrictions were lifted in the UK, the lasting impacts of these restrictions on mental health and PA and on fathers’ childcare roles within families may have shaped the fathers’ responses to the survey.

The findings of our study have important practical and policy implications. It is vital that policy is developed to include and support fathers at this critical life stage for them and their families. The findings from this research suggest that there is scope for designing community-based PA programmes (including those associated with sport and other cultural activities) as settings for engaging new fathers and promoting their mental health, using such programmes as sites for building trusting and meaningful relations between like-minded fathers, and for supporting their greater access to relevant mental health services. Indeed, community programmes that are co-produced with and peer-led by men with lived experience have previously been shown to be effective in helping to develop trust and enable men to engage in PA for mental health benefit [42,43,44]. Further research is needed to explore the potential effectiveness of these types of programmes for new fathers’ mental health, including studies with a more diverse sample of fathers with more diverse experiences than those reported here, if we are to better meet the mental health and PA needs of fathers during the transition into parenthood.

## Figures and Tables

**Table 1:** Participant characteristics.

Characteristic	N	%
**Age**		
25–29 years	1	3.1
30–34 years	12	37.5
35–39 years	13	40.6
40–44 years	6	18.8
**Ethnicity**		
White British	20	62.5
White English	7	21.9
White Scottish	1	3.1
White Welsh	1	3.1
Indian	2	6.3
Chinese	1	3.1
**Employment**		
Full-time	30	93.8
Self-employed	2	6.3
**Family type**		
Opposite-sex married couple	26	81.3
Same-sex married couple	1	3.1
Cohabiting couple	4	12.5
Lone parent	1	3.1
**Disability**		
No	32	100
**Experience of maternal/paternal mental illness**		
Yes: in the past	2	6.3
Maybe past: undiagnosed	7	21.9
Yes: currently	0	0
Maybe current: undiagnosed	2	6.3
No	21	65.6

## Data Availability

The datasets presented in this article are not readily available because of ethical restrictions. Requests to access the datasets should be directed to the corresponding author.
